# Sarcopenia as a prognostic factor in patients with recurrent pancreatic cancer: a retrospective study

**DOI:** 10.1186/s12957-020-01981-x

**Published:** 2020-08-22

**Authors:** Teruhisa Sakamoto, Takuki Yagyu, Ei Uchinaka, Kozo Miyatani, Takehiko Hanaki, Kyoichi Kihara, Tomoyuki Matsunaga, Manabu Yamamoto, Naruo Tokuyasu, Soichiro Honjo, Yoshiyuki Fujiwara

**Affiliations:** Division of Gastrointestinal and Pediatric Surgery, Department of Surgery, School of Medicine, Tottori University Faculty of Medicine, 36-1 Nishi-cho, Yonago, 683-8504 Japan

**Keywords:** Recurrent pancreatic cancer, Sarcopenia, Prognosis

## Abstract

**Background:**

Sarcopenia is a prognostic factor in various cancers. However, the impact of sarcopenia in patients with recurrent pancreatic cancer remains unclear. This study evaluated the prognostic significance of sarcopenia in patients with recurrent pancreatic cancer.

**Methods:**

Seventy-four patients who developed postoperative recurrence of pancreatic cancer after undergoing pancreatectomies were enrolled. Sarcopenia in these patients was defined according to the psoas muscle index (PMI) measured via computed tomography at the third vertebra.

**Results:**

The mean PMIs at the time of recurrence were 4.47 ± 1.27 cm^2^/m^2^ for men and 3.26 ± 0.70 cm^2^/m^2^ for women. Of the 74 patients, 65 (87.8%) were diagnosed with sarcopenia with low PMI. The 2-year post-recurrence survival curve in the sarcopenia group was significantly worse than that in the non-sarcopenia group (*P* = 0.034). Multivariate analysis revealed that sarcopenia at the time of recurrence was an independent prognostic factor (*P* = 0.043) along with a high neutrophil-to-lymphocyte ratio (*P* = 0.004), early recurrence (*P* = 0.001), and chemotherapy after recurrence (*P* = 0.005) in patients with recurrent pancreatic cancer. Furthermore, the area under the curve (AUC) of the combination of sarcopenia and time to recurrence for predicting 2-year survival was 0.763, which was much higher than that of sarcopenia alone (AUC = 0.622).

**Conclusions:**

Sarcopenia is a useful prognostic factor in patients with recurrent pancreatic cancer. The combination of sarcopenia and time of recurrence may more accurately predict post-recurrence survival than can sarcopenia alone.

## Introduction

Prognosis in cancer patients is multifactorial. Tumor-specific factors and individual patient characteristics are risk factors for prognosis in patients with various malignant diseases. Many studies have reported that evaluating the body composition can help determine survival outcomes in cancer patients [1–4].

In 1989, Rosenberg first described sarcopenia as an age-related decrease in skeletal muscle mass [5]. Sarcopenia was later defined as a syndrome characterized by progressive and generalized loss of skeletal muscle mass and strength with adverse outcomes, including physical disability, poor quality of life, and death. Sarcopenia was further classified into primary sarcopenia, caused only by aging, and secondary sarcopenia, with causes such as physical hypoactivity, malnutrition, inflammatory disease, the endocrine system, and malignancy [6, 7]. Previous reports suggest that sarcopenia in cancer patients might reflect hypercatabolism of the skeletal muscle promoted by aggressive biological behaviors from cancer [8–10]. In cancers of the digestive system, sarcopenia is generally recognized as an accurate and quantitative marker of frailty and as a prognostic risk factor [11–15].

Pancreatic cancer remains the cancer with the poorest prognosis among gastrointestinal malignancies despite advanced surgical techniques, development of perioperative treatment, and progress in systemic therapies such as chemotherapy [16, 17]. Preoperative sarcopenia is closely associated with both preoperative cancer stage and poor prognosis in pancreatic cancer [18–20]. Sarcopenia is a prognostic factor in patients with advanced pancreatic cancer because it impairs the response to chemotherapy [21, 22]. However, the impact of sarcopenia for prognosis after recurrence in patients who underwent pancreatectomies for pancreatic cancer remains unclear.

Therefore, this study examined the prognostic significance of sarcopenia in patients with recurrent pancreatic cancer using the psoas muscle index (PMI), a comparatively easy method for representing skeletal muscle volume among the measurements used to evaluate sarcopenia [23].

## Patients and methods

### Patients

We retrospectively reviewed the medical records of 114 patients who were histopathologically diagnosed with pancreatic ductal adenocarcinoma and had undergone pancreatectomies with regional lymphadenectomy at Tottori University Hospital between January 2005 and February 2018. Of these patients, 74 had developed postoperative recurrence as of March 2020 and were enrolled in this study. All patients in this study were of Japanese ethnicity.

Patients were diagnosed with postoperative recurrence via diagnostic imaging, which was performed periodically and included ultrasonography, computed tomography, magnetic resonance imaging, and if needed positron emission tomography, and via the tumor marker, carbohydrate antigen 19-9. Causes of death and patterns of recurrence were determined by reviewing medical records or by direct inquiry with family members. Data on the pathological findings of specimens obtained from the initial pancreatic resection, such as primary tumor size, localization, lymph node metastasis, and histological grading, were collected as per the 8th edition of the Union for International Union Against Cancer classification system of tumor node metastasis [24]. Clinical characteristics, such as age at time of recurrence, sex, chemotherapy after recurrence, time to recurrence after initial surgery, carbohydrate antigen 19-9, and neutrophil-to-lymphocyte ratio as an inflammatory indicator, were obtained from patients’ medical records. Early recurrences were defined as recurrences within 12 months after the initial surgery; late recurrences were those occurring later than 12 months after the initial surgery [25].

### Assessment of PMI and definition of sarcopenia

PMI was calculated according to a previous report [23]. Briefly, to determine the PMI value, computed tomography images at the time of recurrence were analyzed using SYNAPSE VINCENT (Fujifilm, Tokyo, Japan). The cross-sectional areas of bilateral psoas muscles at the third lumbar vertebra (L3) were measured by manual tracing. Then, the PMI at the time of recurrence was calculated by normalizing the cross-sectional areas of bilateral psoas muscles to the height (cm^2^/m^2^). Three authors measured the cross-sectional areas of bilateral psoas muscles at the third lumbar vertebra (L3), and the mean value was used for the analyses.

Sarcopenia was defined as a PMI < 6.36 cm^2^/m^2^ for men and < 3.92 cm^2^/m^2^ for women [23].

### Statistical analysis

To evaluate between-group differences, chi-square or Fisher’s exact probability tests were used for categorical variables, and the Mann–Whitney *U* test was used for continuous variables. Post-recurrence survival curves were calculated using the Kaplan–Meier method, and differences among survival curves were estimated with log-rank tests. Univariate and multivariate analyses with Cox proportional hazards models were performed to determine the significant prognostic factors for overall survival after recurrence. The areas under the curves (AUCs) with respect to predicting 2-year survival were determined using receiver operating characteristic analyses. Variables with *P* < 0.2 were included in the multivariate analysis. *P* values < 0.05 were considered statistically significant. All statistical analyses were conducted using the SPSS software (SPSS for Windows version 24; IBM Corp., Armonk, NY, USA).

## Results

The median follow-up time after recurrence was 10.5 (range 0.3–84.5) months for all patients in this study. Fifty-seven of 74 patients (77.0%) with recurrence received chemotherapy. The first-line chemotherapy was performed as follows: 42 patients received a gemcitabine-based regimen (gemcitabine alone or gemcitabine plus S-1 or gemcitabine plus nab-paclitaxel), 13 received S-1 alone, 1 received uracil-tegafur, and 1 received modified FOLFIRINOX. Second-line chemotherapy was administered to 17 of 57 patients (29.8%) who experienced disease progression. The other patients, except those who received chemotherapy, underwent best supportive care.

The mean PMIs at the time of recurrence were 4.47 ± 1.27 cm^2^/m^2^ for men and 3.26 ± 0.70 cm^2^/m^2^ for women, which were both lower than the sex-specific cutoff values for the PMI used as criteria for sarcopenia in Asian adults [23]. Of the 74 patients with recurrent pancreatic cancer, 65 (87.8%) had low PMIs and were diagnosed with sarcopenia based on these criteria.

Table 1 shows the correlations of the clinicopathological characteristics between patients with low PMI (the sarcopenia group) and those with normal PMI (the non-sarcopenia group). Body mass index at the time of recurrence and lymphatic vessel invasion were significantly correlated between the two groups.

**Table 1:** Clinicopathological characteristics of recurrent pancreatic cancer patients with sarcopenia and without sarcopenia

Characteristics	Sarcopenia group (*n* = 65)	Non-sarcopenia group (*n* = 9)	*P* value
Age at time of recurrence, year, median (range)	73.0 (44–85)	68.0 (53–80)	0.529
Sex, male (*n*, %)	44 (67.7%)	5 (55.6%)	0.476
Body mass index at the time of recurrence, median (range), kg/m^2^	18.9 (13.3–26.8)	23.4 (19.3–27.9)	< 0.001
Primary tumor location (pancreatic head, %)	42 (64.6%)	6 (66.7%)	1.000
Primary tumor size, median (range), mm	28.0 (5–60)	30.0 (25–35)	0.466
Lymph node involvement (present, %)	45 (69.2%)	7 (77.8%)	0.716
Histological grading (G1^a^, %)	30 (46.2%)	6 (66.7%)	0.302
Time to recurrence after pancreatectomy (< 12 months, %)	25 (38.5%)	5 (55.6%)	0.471
Lymphatic vessel invasion (present, %)	23 (35.9%)	7 (77.8%)	0.027
Blood vessel invasion (present, %)	27 (41.5%)	6 (66.7%)	0.176
Perineural invasion (present, %)	9 (13.8%)	2 (22.2%)	0.615
Initial site of recurrence (distant, %)	41 (63.1%)	5 (55.6%)	0.722
Chemotherapy after recurrence (present, %)	49 (75.4%)	8 (88.9%)	0.675
Neutrophil-lymphocyte ratio, median (range)	1.94 (0.36–18.60)	1.62 (0.26–2.63)	0.378
Serum CA19-9 levels at time of recurrence, median (range), U/ml	206.5 (0.1–20,460)	92.1 (10.2–1843)	0.669

Continuous variables are expressed as the median and range

*CA19-9* carbohydrate antigen 19-9

^a^G1, well-differentiated

**Fig. 1:**
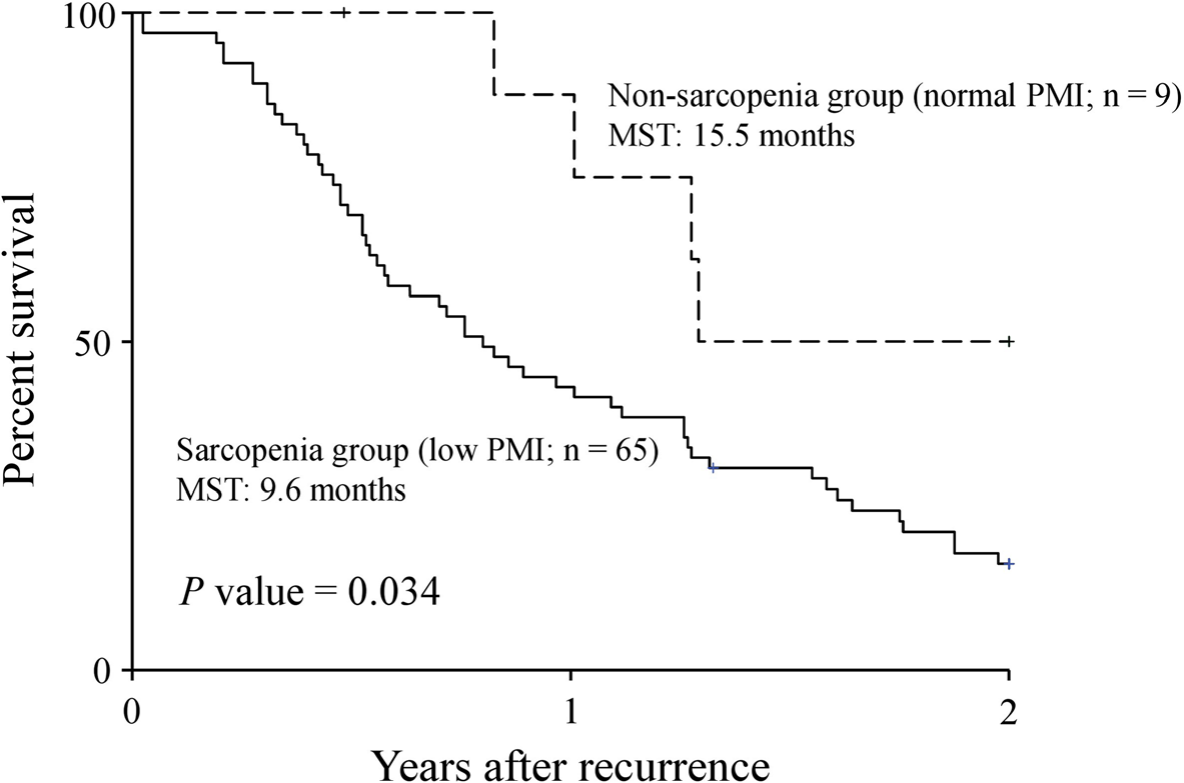
Two-year post-recurrence overall survival curves in recurrent pancreatic cancer patients. Patients with low PMI (sarcopenia group) and with normal PMI (non-sarcopenia group) are compared. PMI, psoas muscle index; MST, median survival time

Figure 1 shows the post-recurrence survival curves for sarcopenia at the time of recurrence. The 2-year post-recurrence survival rates and median survival times after recurrence in patients with sarcopenia were 16.2% and 9.6 months, respectively, which were significantly worse than those in patients without sarcopenia (50.0%, 15.5 months, respectively; *P* = 0.034).

Multivariate analysis revealed that sarcopenia at the time of recurrence was an independent prognostic factor (hazard ratio [HR] 3.094, *P* = 0.043), along with the neutrophil-to-lymphocyte ratio (HR 2.357, *P* = 0.004), time to recurrence after pancreatectomy (HR 2.940, *P* = 0.001), and chemotherapy after recurrence (HR 0.348, *P* = 0.005) in patients with recurrent pancreatic cancer (Table 2).

**Table 2:** Univariate and multivariate analyses of prognostic factors for overall survival among patients with recurrent pancreatic cancer

Variables	Univariate analysis			Multivariate analysis		
	HR	95% CI	*P* value	HR	95% CI	*P* value
Age at time of recurrence (≥ 75 vs. < 75)	0.886	0.521–1.507	0.655			
Sex (male vs. female)	0.875	0.505–1.515	0.633			
BMI at the time of recurrence (< 21.8 kg/m^2^ vs. ≥ 21.8 kg/m^2^)	2.249	1.186–4.264	0.013	1.203	0.608–2.380	0.596
Smoking status at the time of recurrence (current or former vs. never)	0.903	0.478–1.707	0.753			
Primary tumor location (head vs. body and tail)	0.876	0.512–1.499	0.630			
Primary tumor size (≥ 28.9 mm vs. < 28.9 mm)	1.396	0.826–2.361	0.213			
Histological grading for primary tumor (G1 vs. other)	0.729	0.435–1.222	0.230			
Lymph node metastasis at initial surgery (present vs. absent)	1.624	0.911–2.895	0.100	1.560	0.819–2.972	0.176
Surgical margin at the initial resection (present vs. absent)	0.940	0.445–1.986	0.871			
Serum CA19-9 level at time of recurrence (≥ 107.95 U/ml vs. < 107.95 U/ml)	0.931	0.548–1.583	0.793			
NLR (≥ 1.69 mm vs. < 1.69 mm)	2.544	1.460–4.434	0.001	2.357	1.314–4.228	0.004
Time to recurrence after pancreatectomy (< 12 months [early] vs. ≥ 12 months [late])	2.618	1.489–4.604	0.001	2.940	1.551–5.573	0.001
Initial site of recurrence (distant vs. local)	1.227	0.717–2.100	0.456			
Chemotherapy after recurrence (present vs. absent)	0.489	0.266–0.898	0.021	0.348	0.165–0.732	0.005
Sarcopenia at time of recurrence (present vs. absent)	2.846	1.029–7.871	0.044	3.094	1.035–9.248	0.043

*HR* hazard ratio, *CI* confidence interval, *G1* well-differentiated, *BMI* body mass index, *CA19-9* carbohydrate antigen 19-9, *NLR* neutrophil-to-lymphocyte ratio

Using sarcopenia and time of recurrence after pancreatectomy, which were independent prognostic factors, we divided the combination of sarcopenia and time of recurrence into three groups as follows: non-sarcopenia and late recurrence (group A; *n* = 5), non-sarcopenia and early recurrence or sarcopenia and late recurrence (group B; *n* = 29), and sarcopenia and early recurrence (group C; *n* = 40).

**Fig. 2:**
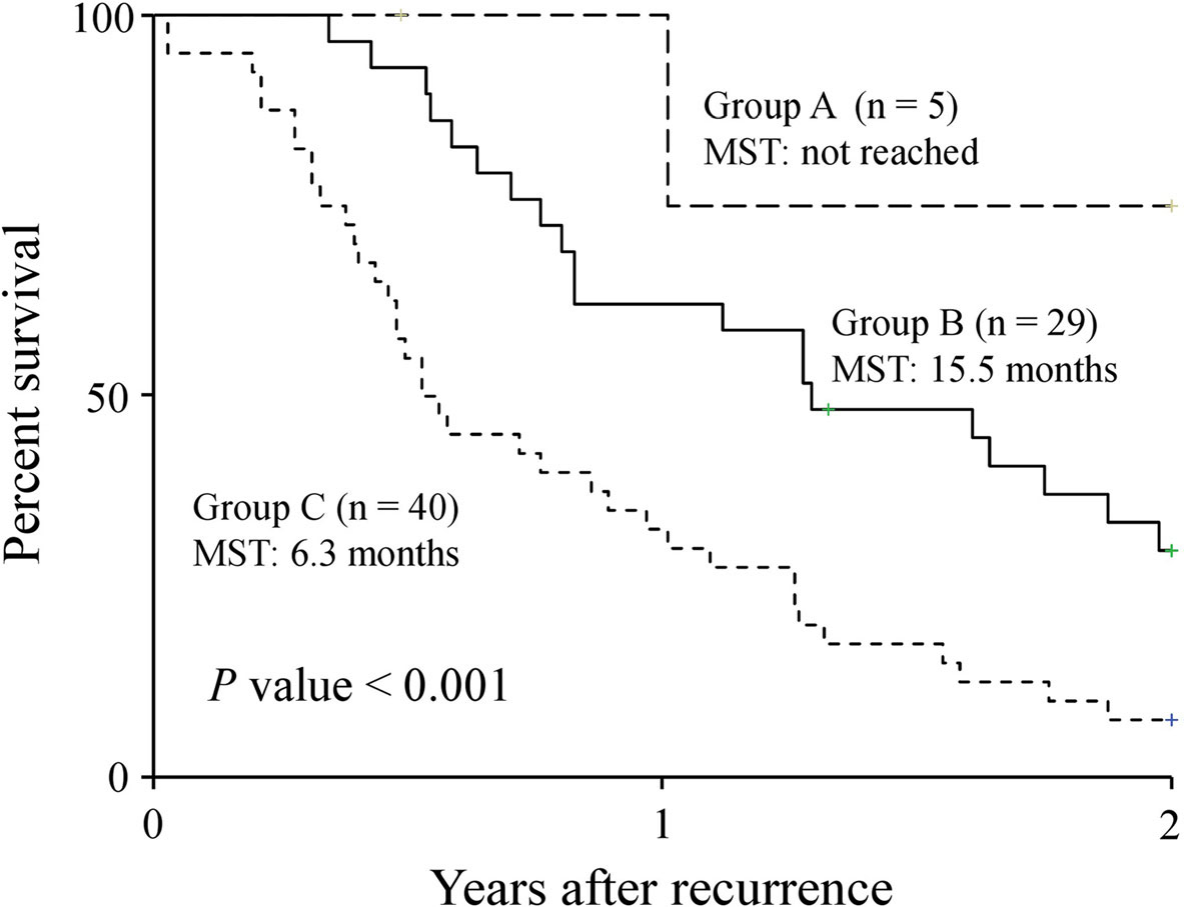
Two-year post-recurrence overall survival curves. Curves are according to the combination of sarcopenia at the time of recurrence after pancreatectomy and time of recurrence. Group A, non-sarcopenia and late recurrence; group B, non-sarcopenia and early recurrence or sarcopenia and late recurrence; group C, sarcopenia and early recurrence. MST, mean survival time

**Fig. 3:**
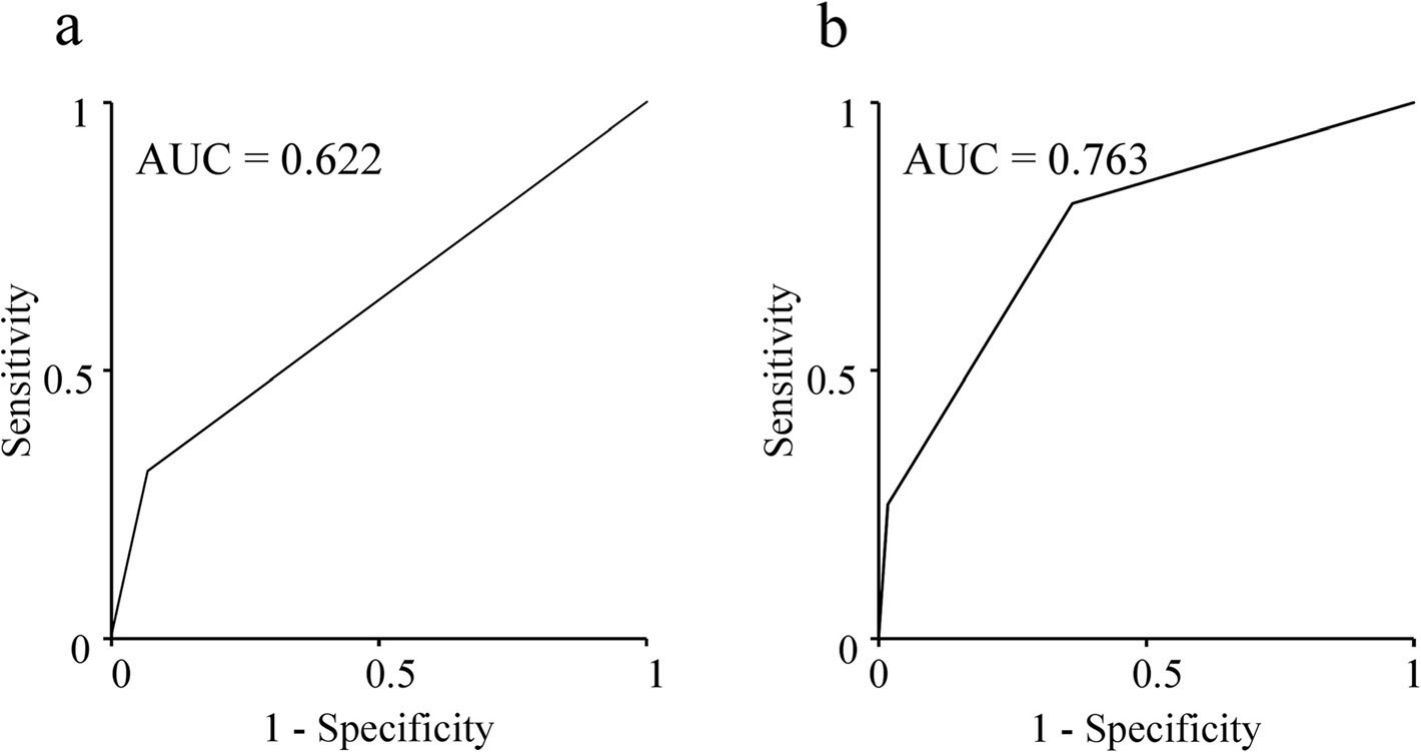
Receiver operating characteristic curves and AUCs of sarcopenia (**a**) and the combination of sarcopenia and time of recurrence after pancreatectomy (**b**) for predicting 2-year survival after recurrence of pancreatic cancer. AUC, area under the curve

The 2-year post-recurrence survival rates were 75.0%, 29.7%, and 7.5% in groups A, B, and C, respectively. The median survival times after recurrence differed significantly at 15.5 months for group B and 6.3 months for group C and were unmet in group A (*P* < 0.001, Fig. 2). The AUC of the combination of sarcopenia and time of recurrence for predicting 2-year survival was 0.763 (Fig. 3a), which was much higher than that of sarcopenia alone (0.622, Fig. 3b).

Thus, the combination of sarcopenia and time of recurrence was more useful than sarcopenia alone for predicting the prognoses of patients with recurrent pancreatic cancer.

## Discussion

This study showed that sarcopenia at the time of recurrence was an independent, unfavorable prognostic factor in patients with recurrent pancreatic cancer. Additionally, the combination of sarcopenia at the time of recurrence and time to recurrence after pancreatectomy more accurately predicted post-recurrent prognoses in patients who underwent surgery for pancreatic cancer than did sarcopenia alone. These findings indicating an association with prognosis after recurrence in patients with recurrent pancreatic cancer are of great importance because few studies have evaluated this issue.

Skeletal muscle loss negatively affects the outcomes of various diseases such as chronic diseases and many cancers [11–15, 26, 27].

In pancreatic cancer, preoperative skeletal muscle loss is closely correlated with overall survival [18–20]. However, the prognostic significance of skeletal muscle loss, that is, sarcopenia, at the time of recurrence has remained unclear. In the present study, skeletal muscle loss at the time of recurrence was a significant prognostic factor in patients with recurrent pancreatic cancer.

The mechanism for the association between skeletal muscle loss and prognosis in cancer patients remains unclear. Skeletal muscle is the largest organ in the human body, accounting for more than 40% of the adult human body weight [9]. Skeletal muscle is also a secretory organ, and accumulating data have shown that muscle cells produce and secrete several hundreds of cytokines and other peptides, termed myokines, which influence various systemic responses [28].

Among numerous myokines, myostatin, a member of the transforming growth factor-β (TGF-β) superfamily, is a key regulator of skeletal muscle mass. Myostatin inactivation increases skeletal muscle volume. In contrast, activated myostatin decreases skeletal muscle volume [29]. TGF-β is a multifunctional regulatory factor and responds to tumor cells by regulating tumor development by suppressing epithelial cell tumorigenesis early in the carcinogenic process via the TGF-β-SMAD pathway. In advanced stages, however, TGF-β switches to promote tumor progression. Increased TGF-β expression by tumor cells promotes tumor progression by enhancing migration, invasion, and survival of tumor cells by stimulating extracellular matrix deposition and tissue fibrosis, perturbing immune and inflammatory functions, and stimulating angiogenesis [30, 31]. Therefore, high TGF-β expression can promote muscle wasting and tumor progression in late-stage cancer patients. In pancreatic cancer, TGF-β plays a paradoxical role by both suppressing and promoting tumors. Previous studies showed that patients with unresectable pancreatic cancer had increased soluble TGF-β; thus, TGF-β was identified as a prognostic factor [32, 33].

Proinflammatory cytokines, such as interleukin (IL)-1, IL-6, and tumor necrosis factor alpha (TNF-α), cause skeletal muscle proteolysis by suppressing muscle genes and activating ubiquitin-proteasome-mediated proteolysis and are closely associated with both sarcopenia and cancer-related cachexia [34].

A strong relationship exists between skeletal muscles and the immune system. Skeletal muscle contains more leukocytes, such as CD8+ T cells, regulatory T cells, and neutrophils, which play important roles in antitumor immunity and are the same as those in the total blood of human adults [35]. Hence, skeletal muscle wasting induces decreased leukocyte numbers and impairs tumor immunity in cancer patients with sarcopenia, which in turn results in tumor progression. Therefore, these relationships may explain the correlation between sarcopenia at the time recurrence and poor prognoses in the current study.

Post-recurrence overall survival in an early recurrence group after curative surgery for pancreatic cancer was reported to be significantly worse than that of the late recurrence group [25]. In that study, larger tumor sizes, high preoperative serum carbohydrate antigen 19-9 levels, poor differentiation on histological grading, presence of microscopic lymphovascular invasion, and highly positive lymph node ratios were reported as risk factors for early recurrence. Another study found more distant metastasis in patients whose pancreatic cancer recurred within 12 months [36]. These findings suggest that patients with early recurrence already had more aggressive tumor biology that led to shorter recurrence-free survival after the initial surgery and a more rapid progression to death. In our study, both sarcopenia and early recurrence were independent prognostic factors in patients whose pancreatic cancer recurred. Sarcopenia is regulated by factors related to patients’ physical and functional conditions, whereas time to recurrence is regulated by tumor behavioral factors. We hypothesized that the combination of these two factors regulated by different factors might better predict the prognosis in patients with recurrent pancreatic cancer than can sarcopenia alone. The combination of sarcopenia and time to recurrence predicted the prognosis in patients with recurrent pancreatic cancer more accurately than did sarcopenia alone.

This study had several limitations. First, it was retrospective with a small sample size and was conducted in a single institution, which could generate bias. No standard methods exist to precisely evaluate sarcopenia because skeletal muscle mass and strength are influenced considerably by ethnicity, body size, age, lifestyle, and cultural background. Although the criteria for sarcopenia using PMI measurement as reported by Hamaguchi et al. were used to evaluate sarcopenia in this study, these criteria were determined from data from healthy Asian young-adult donors for living donor liver transplantation. Furthermore, the participants in this study were all of Asian ethnicity. These facts limit the generalizability of our results; therefore, the optimal PMI cutoff value generalizing the definition of sarcopenia in patients with recurrent pancreatic cancer remains unclear. A large-scale prospective study is needed to verify our results. Additionally, further studies should be conducted to define sarcopenia corresponding to patients’ various conditions to more accurately evaluate sarcopenia-related events.

In conclusion, sarcopenia is a useful prognostic factor in patients with recurrent pancreatic cancer. The combination of sarcopenia and time to recurrence might more accurately predict post-recurrence survival than can sarcopenia alone. Not only systemic chemotherapy but also regular exercise and nutritional therapy after curative surgery might be needed to improve the prognosis in patients with recurrent pancreatic cancer.

## Data Availability

The datasets used and analyzed during the current study are available from the corresponding author on reasonable request.

## Abbreviations

AUC: Area under the curve
HR: Hazard ratio
IL: Interleukin
MST: Median survival time
PMI: Psoas muscle index
TNF-α: Tumor necrosis factor alpha
